# Research on Target Localization Method for Underwater Robot Based on the Bionic Lateral Line System of Fish

**DOI:** 10.3390/biomimetics10090593

**Published:** 2025-09-05

**Authors:** Xinghua Lin, Enyu Yang, Guozhen Zan, Hang Xu, Hao Wang, Peilong Sun

**Affiliations:** 1 School of Mechanical Engineering, Tianjin University of Science and Technology, Tianjin 300457, China; 15175638078@139.com (E.Y.); 15053093367@163.com (G.Z.); xuhxuh0112@163.com (H.X.); wxzy9420@163.com (H.W.); spl1920@163.com (P.S.); 2 Tianjin Key Laboratory of Integrated Design and Online Monitoring of Mechanical Equipment for Light Industry and Food Engineering, Tianjin 300457, China

**Keywords:** underwater robot, bionic lateral line, flow field sensing, target localization, tracking navigation

## Abstract

This paper is based on the fish lateral line sensing mechanism and aims to determine the coupling relationship between the flow field sensing signal and target source position information. Firstly, according to the flow field distribution characteristics of the target source, the equivalent multipole model of the flow field disturbance during the underwater motion of the SUBOFF model is constructed, and then the target localization function based on the least squares method is established according to the theory of potential flow, and the residual function of the target localization is solved optimally using the quasi-Newton method (QN) to obtain the estimated position of the target source. On this basis, a curved bionic lateral line sensing array is constructed on the surface of a robotic fish, and the estimated location of the target source is obtained. The curvilinear bionic lateral line sensing array is constructed on the surface of the robotic fish, and the effectiveness and robustness of the above localization methods are analysed to validate whether the fish lateral line uses the pressure change to sense the underwater target.

## 1. Introduction

The environment perception technology of underwater robots has received increased attention in ocean engineering applications, because it is not only related to the conventional functions of AUVs (Autonomous Underwater Vehicles), such as autonomous navigation and obstacle avoidance, but it also directly affects operational tasks such as underwater target detection and topographical and geomorphological measurements, and it has become a key technology that all countries in the world are competing to develop. At present, underwater perception technology mainly relies on acoustic, optical, and electromagnetic signals [1]. For example, Ge Xiyun et al. proposed a robust positioning algorithm that combines distributed estimation and particle swarm optimisation to address positioning errors caused by underwater acoustic communication and sampling uncertainty [2,3,4]. Tang Guoqiang et al. proposed a submarine robot visual positioning system that utilises a three-dimensional AprilTag structure, high-resolution cameras, and fusion filtering to improve multi-label transition performance, and experiments verified its stability and applicability in complex situations [5].

Acoustic signals have become the most commonly used perception method in underwater target tracking due to the fact that they are not easily absorbed in seawater, the existence of a long propagation distance, and the stability of the signals [6,7,8], but the current rapid development of underwater anechoic technology in all countries of the world has made the limitations of acoustic systems in tasks such as covert detection and target tracking even more pronounced. For example, since 2017, the United States has been actively developing a paint-like ‘superhydrophobic’ coating that can absorb 91% of incoming acoustic energy and has a reflectivity of less than 3%; the effect is very significant. Paris Diderot University in France and other units have also carried out research on the application of tiny bubbles in thin coatings, so that the noise level of the underwater vehicle is infinitely close to the ambient noise. Close to the environmental noise, relying on acoustic systems is insufficient to meet detection needs. In addition, acoustic systems are prone to problems such as ghosting and ambient reverberation due to the multi-pathway effect in networked detection, while their excessive price and power consumption make the cost of the whole network system increase significantly [9,10]. Optical signals can acquire a large amount of environmental detail in real time, with ultra-high transmission bandwidth, and are widely used in underwater target tracking. In recent years, great progress has been made in the fields of image enhancement techniques and feature recognition algorithms [11,12,13]. However, the inherent characteristics of being susceptible to object occlusion and conditions such as light and water quality make it impossible to effectively compensate for its limitations in underwater target detection and tracking. Underwater electromagnetic sensing is an emerging low-frequency detection technology that is not affected by environmental noise and light conditions, etc., but the high attenuation rate in water is the main factor limiting its application, which needs to be compensated by the placement of a large number of communication nodes, with limitations in terms of infrastructure costs and detection range [14,15]. Therefore, with the increase in AUV near reconnaissance, cooperative clustering, near-bottom navigation and other mission modes, the service environment of AUVs has become complex, leading to an urgent demand for the near-field target detection and positioning of AUVs; however, it is difficult to provide AUVs with ideal near-field target sensing information with the existing technology, and it has become a technological bottleneck that restricts the future development of underwater environment sensing, detection, tracking, and navigation technology, and it is necessary to explore new underwater near-field targets. The exploration of new underwater near-field target positioning and tracking technology is very necessary.

**Figure 1:**
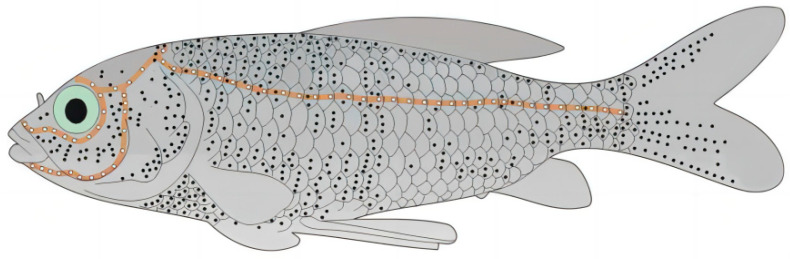
Schematic distribution of lateral lines in goldfish.

Fish have developed a unique organ for tactile perception over a long period of evolutionary time—the lateral line system; this has the function of sensing water velocity and water pressure, and can achieve information interaction, prey tracking, map positioning, manoeuvring to avoid obstacles, target recognition and other functions after transmitting the signals from the sensed flow field to the central system [16,17,18], which provides a bionic mechanism reference for the development of a new type of underwater sensing technology. The earliest research began in the field of biology, with the biologists involved in the mid-17th century having already discovered the presence of these transparent mucus tubes within the epidermis of fish. Figure 1 shows a schematic diagram of the lateral line of goldfish [19,20]; the lateral line tubes are mainly distributed on both sides of the fish trunk, through the entire axial length, and are more densely distributed in the head. As shown by the white dots in the figure for the pipeline on the interval of the small holes, these open air holes in the lateral line are called the Canal Lateral Line (CLL). In addition, another kind of lateral line is directly exposed to a cilia-type structure in the fluid, such as that shown in the black dots in the figure; this kind of lateral line is called the “Superficial Lateral Line” (SLL).

Both CLL and SLL consist of basic sensory units called ‘neural mounds’, which are mainly composed of two parts: the glial roof and the hair cells [21]; one of the gelatinous tops is filled with a gel-like substance that wraps around the outer layer of the hair cell and serves to protect it and transmit deformations. Hair cells are the main sensory tissue in the neural thalamus and are topped by ciliated bundles in a stepped distribution, which are divided into two types, kinetic and static cilia, according to the state of movement. The dynamic cilia are longer in structure and fewer in number, whereas the static cilia are shorter and more numerous, and the height of the auto-cilia decreases from one side of the cilium to the other in a stepwise fashion. The main role of the hair cells is to convert the deformation signals of the cilia into neuroelectric signals, and the special stepped distribution state makes the hair cells sensitive to deformation only towards the side of the moving cilium, so the response of the hair cells is unidirectional.

Related studies have shown that the sensory range of fish lateral lines can cover the near-field region, with a higher detection accuracy for low-frequency targets within a distance of the centre (equivalent to the wavelength of underwater sound waves). Therefore, in the future, a fusion perception technology combining sonar and biomimetic lateral line systems could be employed for underwater target detection. For high-frequency target signals, sonar detection would be prioritised, while for near-field targets, the biomimetic lateral line system could capture flow field disturbance signals, thereby addressing the blind spots in sonar technology’s near-field perception capabilities. This approach holds promise as an effective method for achieving low-cost, low-power, strong noise-resistant, and highly covert detection techniques.

Therefore, this paper investigates the flow field perception mechanism based on the fish lateral line system and equivalent multipole model when stabilising heading by constructing the SUBOFF model, establishes a target localization function based on the least squares method, and uses the Quasi Newton (QN) method to optimise the residual function of target localization, thereby obtaining the estimated position of the target source. Additionally, it constructs a bionic lateral line sensing array on the surface of the robotic fish, analyses the effectiveness and robustness of the above-mentioned localization method, and verifies that the fish lateral line perceives underwater targets using pressure changes.

## 2. Target Positioning Theoretical Model Based on Flow Field Perception

### 2.1. Equivalent Dipole Model for Underwater Moving Targets

Hanke discussed in detail the characteristics of various hydrodynamic target sources in lateral line perception in his work [22]. In the lateral line perception environment, targets with volume changes are extremely rare, so their monopole components can be ignored. Therefore, within a certain distance range, the flow field generated by underwater moving targets is very similar to that of a dipole, while the contributions from quadrupoles and higher-order poles can be neglected relative to the dipole source. Thus, the primary feature component perceived by the fish lateral line system is generated by the dipole source, making it a typical excitation source for lateral line perception research [23,24,25,26].

The three-dimensional potential flow field around a moving fish with an axially symmetric body was investigated mathematically by Hassan [27,28,29]. In this paper, we will conduct the reverse research based on these studies; assuming that the distribution characteristics of the flow field around the bionic fish are given, the location method of interference source will be studied. We assumed that the fluid is ideally incompressible in this paper, so the equivalent dipole model of SUBOFF underwater motion can be analysed by applying the method of potential flow theory, and the Navier–Stokes equations describing the motion of the incompressible fluid are shown in Equation (1).

(1) $$\frac{\partial V}{\partial t}+\left(V\cdot \nabla \right)V=f-\frac{\nabla p}{\rho }+\upsilon \cdot \nabla^{2}V$$

In Equation (1), V is the fluid velocity, ρ is the fluid density, p is the pressure, f is the volume force acting on the fluid, which is gravity in this study, and υ is the kinematic viscosity of the fluid. When fluid viscosity is neglected, Equations (3)–(9) can be expressed as the Euler equations for inviscid fluid motion, which are represented in the Cartesian coordinate system, as shown in Equation (2).

(2) $$\left\{\begin{array}{l} \rho \left(\frac{\partial u}{\partial t}+u\frac{\partial u}{\partial x}+v\frac{\partial u}{\partial y}+w\frac{\partial u}{\partial z}\right)=\rho \cdot f_{x}-\frac{\partial p}{\partial x}\\ \rho \left(\frac{\partial v}{\partial t}+u\frac{\partial v}{\partial x}+v\frac{\partial v}{\partial y}+w\frac{\partial v}{\partial z}\right)=\rho \cdot f_{y}-\frac{\partial p}{\partial y}\\ \rho \left(\frac{\partial w}{\partial t}+u\frac{\partial w}{\partial x}+v\frac{\partial w}{\partial y}+w\frac{\partial w}{\partial z}\right)=\rho \cdot f_{z}-\frac{\partial p}{\partial z} \end{array}\right.$$

In practical applications, Equations (3)–(10) can be simplified. When the velocity of fluid motion is very small, the quadratic term of velocity in the equation can be neglected, and the volume force is gravity. Within the considered spatial range, the volume force can be regarded as a constant with a component only in the vertical direction, and the above equation can be simplified to Equation (3).

(3) $$\left\{\begin{array}{l} \rho \left(\frac{\partial u}{\partial t}\right)=-\frac{\partial p}{\partial x}\\ \rho \left(\frac{\partial v}{\partial t}\right)=-\frac{\partial p}{\partial y}\\ \rho \left(\frac{\partial w}{\partial t}\right)=\rho \cdot f_{z}-\frac{\partial p}{\partial z} \end{array}\right.$$

In the horizontal plane containing the target source, the vertical fluid velocity is consistently zero. Disregard the third equation in Equation (3). The first two equations are expressed in polar coordinates as Equation (4). Integrating these equations yields the pressure distribution function of the flow field, as demonstrated in Equation (5).

(4) $$\left\{\begin{array}{l} -\frac{\partial p}{\partial r}=\rho \frac{\partial v_{r}}{\partial t}\\ -\frac{\partial p}{r\partial \theta }=\rho \frac{\partial v_{\theta }}{\partial t} \end{array}\right.$$

(5) $$p=-\int \rho \frac{\partial v_{r}}{\partial t}dr+C_{r}=-\int \rho \frac{\partial v_{\theta }}{\partial t}rd\theta +C_{\theta }$$

In the equation, $v_{r}$ is the radial velocity and $v_{\theta }$ is the tangential velocity.

**Figure 2:**
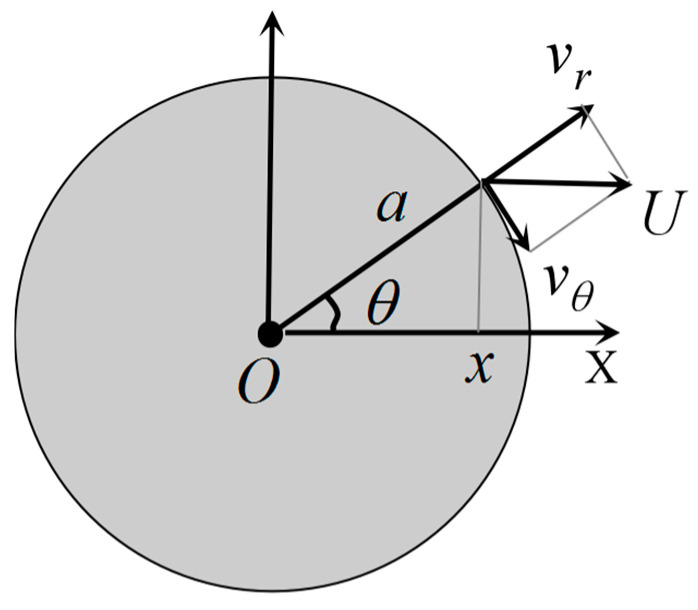
Boundary conditions of the vibrating ball.

If the equilibrium position of the target source centre motion is taken as the coordinate origin and the motion direction as the *x*-axis, as shown in Figure 2, the boundary condition when fluid viscosity is neglected is a slip condition, i.e., the normal velocity is continuous. The amplitude s of the target motion is very small relative to the radius *a*, which is *s* << *a*. In this way, the outer surface of the vibrating ball can be considered to be fixed, and therefore the equations for the vibrational displacement, surface normal velocity and centre velocity of motion can be obtained as shown in Equation (6), respectively.

(6) $$\left\{\begin{array}{l} A=s\cdot \sin\left(\omega \cdot t\right)\\ V_{bn}=U\cdot x/r=U\cdot \cos\left(\omega \cdot t\right)\\ U=U_{0}\cdot \cos\left(\omega \cdot t\right)=s\cdot \omega \cdot \cos\left(\omega \cdot t\right) \end{array}\right.$$

For incompressible fluids with irrotational flow, the Laplace equation $\nabla^{2}\phi =0$ can be satisfied. When fluid viscosity is neglected, it can be assumed that the velocity of the fluid and the velocity of the small sphere are equal in the normal direction at the boundary interface. Therefore, the velocity potential function can be expressed as Equation (7).

(7) $$\left\{\begin{array}{l} \frac{\partial \phi }{\partial r}=U\cdot \cos\theta \\ r=a \end{array}\right.\Rightarrow \left\{\begin{array}{l} \phi =A\frac{\partial }{\partial x}\frac{1}{r}=-A\frac{\cos\theta }{r^{2}}\\ U=2A/a^{3} \end{array}\right.\Rightarrow \phi =-\frac{a^{3}}{2r^{2}}\cdot U\cdot \cos\theta$$

In the equation, *a* is the radius of the small sphere. Thus, the radial velocity $V_{r}$ and tangential velocity $v_{\theta }$ of the fluid in the small sphere coordinate system are given by Equation (8), respectively. According to Equation (5), the pressure in the small sphere coordinate system can be obtained as shown in Equation (9). Therefore, the velocity in the small sphere Cartesian coordinate system can be expressed as Equation (10).

(8) $$\left\{\begin{array}{l} v_{r}=\frac{\partial \phi }{\partial r}=\frac{a^{3}}{r^{3}}U\cos\theta \\ v_{\theta }=\frac{1}{r}\frac{\partial \phi }{\partial \theta }=\frac{a^{3}}{2r^{3}}U\sin\theta \end{array}\right.$$

(9) $$p\left(r,\theta ,t\right)=-\frac{\rho \omega a^{3}}{2r^{2}}\cdot U_{0}\cdot \cos\theta \sin\left(\omega \cdot t\right)+C$$

(10) $$\left\{\begin{array}{l} v_{x}=v_{r}\cdot \cos\theta -v_{\theta }\cdot \sin\theta =\frac{a^{3}U}{2}\frac{2{\left(x-x_{s}\right)}^{2}-{\left(y-y_{s}\right)}^{2}}{{\left[{\left(x-x_{s}\right)}^{2}+{\left(y-y_{s}\right)}^{2}\right]}^{5/2}}\\ v_{y}=v_{r}\cdot \sin\theta -v_{\theta }\cdot \cos\theta =\frac{a^{3}U}{2}\frac{3\left(x-x_{s}\right)\left(y-y_{s}\right)}{{\left[{\left(x-x_{s}\right)}^{2}+{\left(y-y_{s}\right)}^{2}\right]}^{5/2}} \end{array}\right.$$

**Figure 3:**
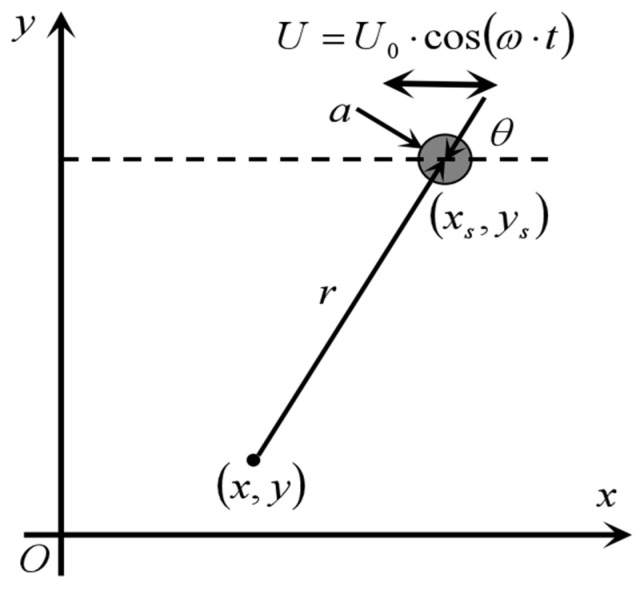
Coordinate system and parameters of the vibrating ball.

The amplitude of the pressure change is extracted according to Equation (9), as shown in Equation (11), and the experimental environment can be reached by making an FFT of the acquired pressure signal and extracting the amplitude at frequency ω. Combined with Figure 3, Equation (11) can be rewritten in the form of a right-angled coordinate, as shown in Equation (12), which can be used for the localization of underwater targets based on the equivalent dipole model.

(11) $$p\left(r,\theta \right)=-\frac{\rho \omega a^{3}}{2r^{2}}U_{0}\cos\theta =-\frac{\rho s\omega^{2}a^{3}}{2r^{2}}\cos\theta$$

(12) $$p\left(x,y\right)=-\frac{\rho \omega a^{3}}{2r^{2}}\cdot \frac{x-x_{s}}{{\left[{\left(x-x_{s}\right)}^{2}+{\left(y-y_{s}\right)}^{2}\right]}^{3/2}}$$

### 2.2. Simulation Validation of an Equivalent Dipole Localization Model

The AUV target source in this paper is the classic SUBOFF submarine 1/10 scale-down model, i.e., the hull length is 436 mm and the diameter is 50.8 mm. In order to simplify the localization method, only the bare hull model of SUBOFF is considered as the localization target in this paper, and the bionic sidereal perceptual arrays are located at the distance of d=10 mm positions below the model, as shown in Figure 1.

**Figure 4:**
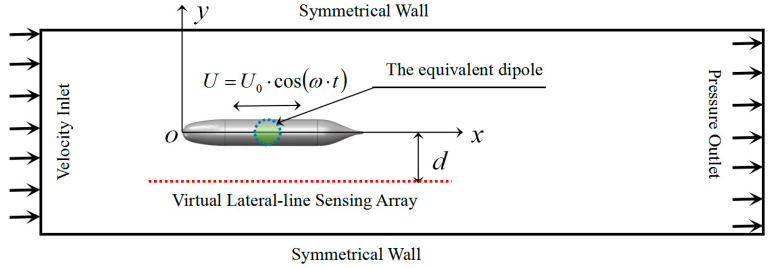
CFD simulation model with boundary conditions.

The computational domain of CFD simulation is a cylindrical structure. In order to simulate the fact that the wake field can be fully developed when the AUV sails in a broad flow field environment, and considering the economy of computational resources, the dimension of the computational domain is set to 5L×12D (L is the total length of the AUV model, and D is the diameter of the cabin of the AUV model), in which the length of the onshore section of the AUV model is L, and the length of the offshore section is 3L. By comparing with the experimental results in the literature [30], it is demonstrated that the above computational domain dimensions can accurately model the flow field distribution around the AUV. In order to create a uniform flow field environment, a velocity inlet of constant size ($u_{x}=U_{\infty }=1,u_{y}=0$) is used as the inlet boundary of the computational domain, a pressure outlet (P=0) is used for the outlet boundary, a symmetric wall ($\partial u_{x}/\partial y=0,u_{y}=0$) is used for the boundary, and the target body surface is used for the no-slip rigid wall condition, as shown in Figure 4.

The pre-processing link in the process of CFD has been introduced in detail in our previous article [31]. In that paper, we carried out the verification of grid size optimisation. The orthographic projected area of the target body $S=2.2\;m^{2}$, the five grid sizes are set as $s_{i}=i\times S/1000$, and the drag coefficient $C_{d}$ generated by the AUV at 20 knots is used as the characterisation parameter. Finally, in order to balance the computational accuracy and computational cost, the first layer of meshes closest to the target body $\chi_{1}=s_{2.0}$ is used.

In this paper, the CFD software of Ansys Fluent 2019 R3 is used to simulate and analyse the flow field disturbance generated by the above AUV model during underwater navigation, in which the turbulence model is described by the modified RNG k-ε turbulence equation, the solution process adopts the non-constant stealth algorithm, and the fluid is assumed to be an incompressible and continuous medium during the simulation. The near-wall treatment method adopts the enhanced wall function to solve the flow at the wall, and the pressure–velocity coupling is solved using the SIMPLEC method, which is discretised by the second-order windward format; a smaller under-relaxation factor is adopted and the convergence determination condition is that the residuals of all calculations are less than 1 × 10^−4^. The drag coefficients on the model are also monitored, and the calculations are considered to have converged after the variation in the drag coefficients lies in the range of 0.001.

In our previous study, the reliability of the above numerical calculation scheme is verified by using the pool towing test data, which was gathered by David Taylor Naval Ship Research and Development Center (USA) [31]. In this paper, the SUBOFF rotary body model with the same size as that in the literature is selected. The literature introduces the results of towing pool experiments, which were conducted by the China Ship Scientific Research Center (CSRC) [30]. The CFD simulation of the above calculation scheme is carried out at the same speed of 10 m/s, and the drag coefficient $C_{d}$ in the experimental data is compared and verified, and the results are shown in Table 1. It can be seen that the numerical results match the experimental results well, where the error is less than 3%.

**Figure 5:**
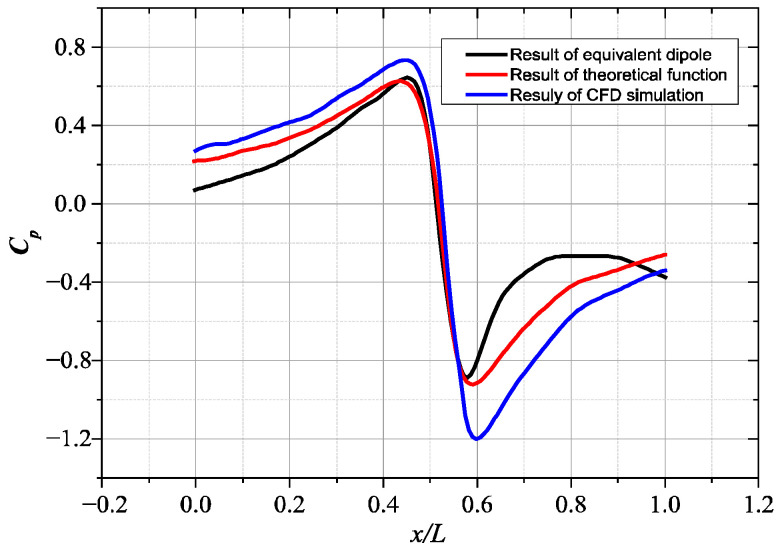
Comparison curve of pressure coefficient distribution on virtual sideline.

The above AUV model is equated to a dipole vibration source and the centre position of the target source is used as the vibration centre of gravity, as shown in Figure 4. By adjusting the vibration frequency and amplitude, the equivalent flow field distribution generated by the original model when sailing at uniform speed can be obtained. In this paper, the pressure signal distribution curve on the sideline sensing array at the 10th second is shown in Figure 5 when the AUV is selected to be sailing underwater at a uniform speed of 3 m/s and the pressure signal distribution curve on the sideline sensing array is collected at the 10th second moment.

It is found that when the diameter *a* of the equivalent dipole model is 50.8 mm, and the vibration frequency of the ball is set to 45 Hz, the vibration direction is parallel to the *x*-axis, and the motion amplitude is 4 mm according to the UDF moving mesh method; the pressure signals generated by the equivalent dipole model match the flow field signals generated by the original SUBOFF model very well, and at the same time, according to Equation (12), we can construct the pressure of the equivalent dipole source model. The actual position of the SUBOFF model can also be estimated well. As shown in Figure 5, the dipole source is therefore representative of the general target and can be used to describe the flow field perturbations acting on the sidestreams in real environments, and the subsequent target localization studies in this paper will replace the centre of the AUV model with an equivalent dipole source.

## 3. Positioning Methods for Equivalent Modelling of Underwater Targets

### 3.1. Least Squares Based Target Location Function

The research objective of this paper is to find the centre coordinates of the target source; the Least Square Method (LS) is used to find the best function match of the data by solving a solution of the residual function so that the sum of the squares of the errors between the estimated results and the real results is minimised; therefore, the role of the LS is to find a target source location $\left(x,y\right)$ such that its theoretical distribution of the pressure field distribution model best matches the pressure distribution obtained from actual measurements, so the estimation of the target location using the least squares method is feasible.

**Figure 6:**
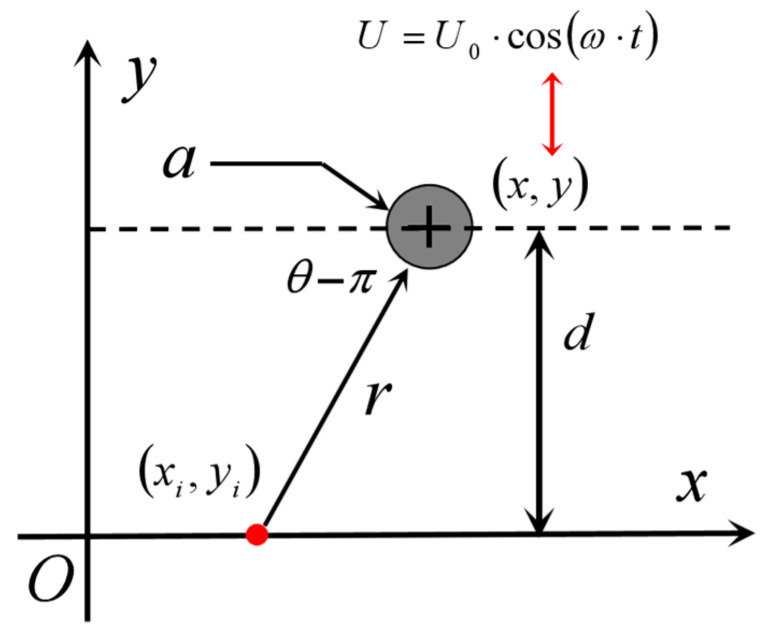
Schematic representation of the coordinates for the positioning of the equivalent model of the underwater target.

The red arrow in Figure 6 shows when the dipole vibrates parallel to the *Y*-axis. The theoretical value of the pressure at each sensor position for the dipole source located at point $\left(x,y\right)$ in the Cartesian coordinate system can be obtained according to the dipole source pressure field distribution model $p\left(x_{i},y_{i}\right)$, as shown in Equation (13). On the basis of the target source pressure field distribution model, using LS to establish the residual function, the residual function in this paper is the residual function of the actual measured pressure value of each measurement point and the target source at $\left(x,y\right)$. We calculated the corresponding theoretical value of the difference between the sum of squares, that is, the definition of the position $\left(x_{i},y_{i}\right)$ at the signal monitoring point $S_{i}$ (shown by the red dot in Figure 3) of the measured value of $M_{i}$. The pressure signal amplitude was obtained from the raw data by Fast Fourier Transform (FFT) and a non-negative objective function $J\left(x,y\right)$ was constructed using LS, as shown in Equation (14).

(13) $$p\left(x_{i},y_{i}\right)=\frac{\rho U_{0}\omega a^{3}\left|y-y_{i}\right|}{{\left[{\left(x-x_{i}\right)}^{2}+{\left(y-y_{i}\right)}^{2}\right]}^{3/2}}$$

In the equation, ρ is the density of water and the angular frequency ω=2πf.

(14) $$\begin{array}{l} J\left(x,y\right)={\sum_{i=1}^{n}\left[M_{i}-p\left(x_{i},y_{i}\right)\right]}^{2}\\ =\sum_{i=1}^{n}{\left\{M_{i}-\frac{\rho U_{0}\omega a^{3}\left|y-y_{i}\right|}{{\left[{\left(x-x_{i}\right)}^{2}+{\left(y-y_{i}\right)}^{2}\right]}^{3/2}}\right\}}^{2} \end{array}$$

This paper uses nine pressure sensors, with nine theoretical values and actual values. Therefore, a residual function optimisation method is used to find a coordinate (x,y) that minimises the value of th eresidual function $J\left(x,y\right)$, so that coordinate (x,y), where $J\left(x,y\right)$ tends towards 0, is the coordinate of the dipole.

### 3.2. Target Positioning Optimisation Algorithm Based on the Quasi-Newton Method

The BFGS algorithm (proposed by Broyden, Flecher, Goldfarb, and Shanno), using the quasi-Newton method, is used to perform unconstrained optimisation of the residual function shown in Equation (14). Based on the positioning method of static spherical targets in our previous research [26], the initial predicted position of the target source $x^{0}$ can be determined by collecting the flow field signals on the bionic lateral line sensing array. Then, the specific position of the equivalent dipole model can be accurately predicted based on the iterative process of BFGS calculation. The iterative process is as follows:

Firstly, we present the iterative equation, as shown in Equation (15):

(15) $$x^{k+1}=x^{k}-\lambda_{k}H_{k}g_{k}$$

In the equation, $\lambda_{k}$ is the iteration step size. If $H_{k}=G_{k}^{-1}$ is set, then Equation (15) becomes the Newton iteration equation. The quasi-Newton method uses the target function value and first-order derivative information to construct a suitable $H_{k}$ to approximate $G_{k}^{-1}$, so that $G_{k}^{-1}$ does not need to be calculated and the algorithm converges quickly.

Select $B_{k+1}$ to satisfy Equation (16), then $B_{k+1}$ can be a good approximation of $G_{k+1}$.

(16) $$B_{k+1}s_{k}=y_{k}$$

The BFGS algorithm is used to correct $B_{k}$ and $H_{k}$, as shown in Equations (17) and (18), where $\omega_{k}$ is shown in Equation (19).

(17) $$B_{k+1}=B_{k}-\frac{B_{k}s_{k}s_{k}^{T}B_{k}}{s_{k}^{T}B_{k}s_{k}}+\frac{y_{k}y_{k}^{T}}{y_{k}^{T}y_{k}}$$

(18) $$\begin{array}{ll} H_{k+1} & =H_{k}-\frac{\left(s_{k}-H_{k}y_{k}\right)s_{k}^{T}+s_{k}{\left(s_{k}-H_{k}y_{k}\right)}^{T}}{s_{k}^{T}y_{k}}-\frac{{\left(s_{k}-H_{k}y_{k}\right)}^{T}y_{k}}{s_{k}^{T}y_{k}}s_{k}^{T}s_{k}\\ & =H_{k}-\frac{H_{k}y_{k}y_{k}^{T}H_{k}}{y_{k}^{T}H_{k}y_{k}}+\frac{s_{k}s_{k}^{T}}{y_{k}^{T}s_{k}}\omega_{k}\omega_{k}^{T} \end{array}$$

(19) $$\omega_{k}:=\sqrt{y_{k}^{T}H_{k}y_{k}}-\left(\frac{s_{k}}{y_{k}^{T}s_{k}}+\frac{H_{k}y_{k}}{y_{k}^{T}H_{k}y_{k}}\right)$$

For the unconstrained optimisation problem $\underset{x\in {ℜ}^{n}}{\min}\;f(x)$, select the initial point $x^{0}\in {ℜ}^{n}$. If $\left\|g_{0}\right\|=0$, the algorithm terminates; otherwise, select the initial matrix $H_{0}$(usually taken as the identity matrix I) and set k:=0.

Step 1: Place $d^{k}:=-H_{k}g_{k}$;

Step2: Calculate the step size $\lambda_{k}$ using precise one-dimensional linear search $f\left(x^{k}+\lambda_{k}d^{k}\right)=\underset{\lambda \geq 0}{\min}f\left(x^{k}+\lambda_{k}d^{k}\right)$;

Step 3: Set $x^{k+1}=x^{k}-\lambda_{k}d^{k}$; if $\left\|g_{0}\right\|=0$, then the algorithm terminates; otherwise, proceed to Step 4.

Step 4: Substitute $s_{k}=x^{k+1}-x^{k}$ and $y_{k}=g^{k+1}-g^{k}$ into Equations (4)–(6) to obtain $H_{k+1}$.

Step 5: After k=k+1, go back to Step 1.

For the optimisation of the residual function of the least squares method based on the quasi-Newton method, use the above BFGS algorithm for iterative solution. Once the preset accuracy is reached, the desired dipole source position $\left(x,y\right)$ is obtained.

## 4. Analysis of Target Positioning Results

### 4.1. Lateral Line Perception Array Model

**Figure 7:**
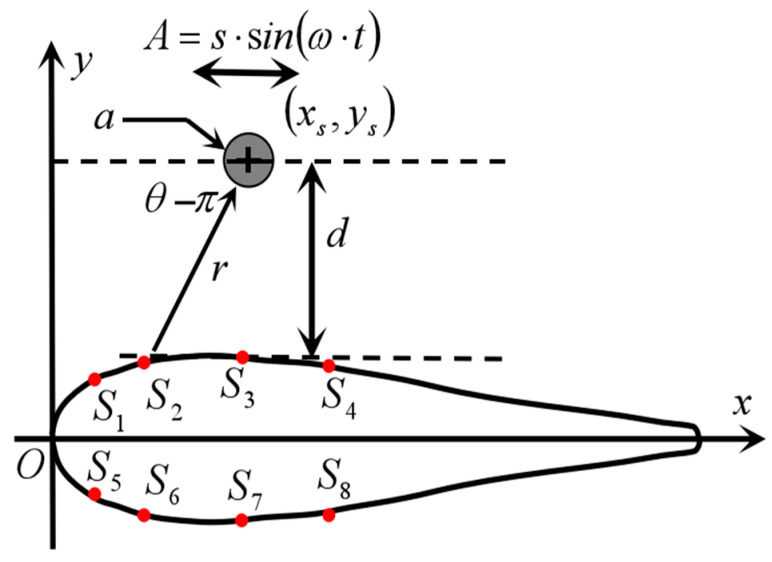
Coordinate definition of the biomimetic robot fish model.

CFD was used to perform flow field simulation calculations on the above model. The two-dimensional coordinates of the virtual robotic fish model with an axially symmetric body are defined as shown in Figure 7. The sensor positions (*S*_1_~*S*_4_) on one side are shown in Table 2. The diameter a of the vibrating ball is 50.8 mm. The vibration frequency of the ball is set to 45 Hz using the UDF dynamic mesh method. The vibration direction is parallel to the *x*-axis, and the amplitude of motion is 10 mm. The computational domain dimensions are L3500 mm × W1000 mm × H1000 mm, and the target model is located 500 mm below the water surface.

**Figure 8:**
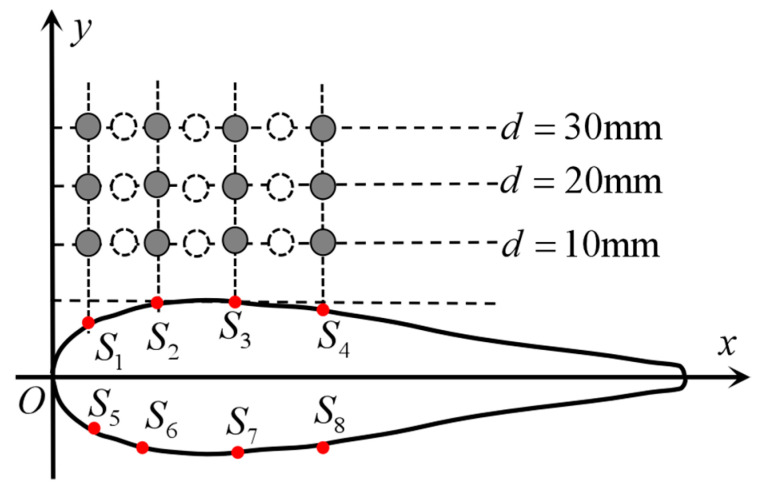
Schematic diagram of data collection locations.

Simulation calculations are divided into three groups based on the distance d between the vibrating ball and sensor *S*_3_, namely *d* = 10 mm, *d* = 20 mm, *d* = 30 mm. In each group of experiments, pressure amplitude data was collected from seven positions: the vibration ball directly facing sensors *S*_1_, *S*_2_, *S*_3_, *S*_4_ and the midpoints of the positions of the two adjacent sensors, as shown in Figure 8.

In summary, the research model of this paper was constructed; this is a virtual robotic fish with an axially symmetric body. The dipole oscillating ball is used as the target model. The sampling CFD numerical calculation method simulates the flow field structure at different positions around the target located around the robotic fish. The amplitude of pressure fluctuations at the pressure monitoring points on the surface of the robotic fish was collected as input signals. Research on methods for locating the coordinates of small ball positions was conducted, with the specific model parameters shown in Table 3.

### 4.2. Error Analysis of Targeting Results

**Figure 9:**
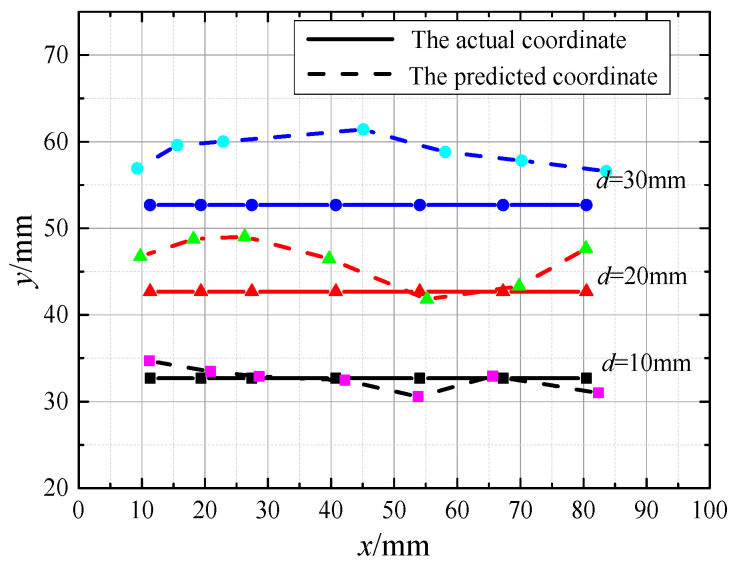
Predicted localization results at different distances.

The equivalent dipoles are set at different positions on the side of the ‘robotic fish sensing sideline’ (three in the vertical position and 7 in the horizontal position), where the true position is recorded as $\left(x_{s},y_{s}\right)$. The pressure data are acquired from nine monitoring points, residual functions are optimised using quasi-Newton methods, and the predicted coordinates of the equivalent dipole $\left(x_{e},y_{e}\right)$ are obtained. The positioning error in the *x*-axis direction is recorded as $\Delta x=x_{e}-x_{s}$. The positioning error in the *y*-axis direction is denoted as $\Delta y=y_{e}-y_{s}$. The distribution curve of the predicted position compared to the actual position is shown in Figure 9.

As can be seen in Figure 9, the prediction results for both *x*-axis coordinates and *y*-axis coordinates at position d=10 mm (identified by the solid square) are in good agreement with the actual positions. The average error in the *x*-axis coordinate prediction is 1.16 mm, with a relative error of only 2.3% with respect to the equivalent dipole diameter; the average error in *y*-axis coordinate prediction is 1.04 mm, with a relative error of only 2.05% with respect to the equivalent dipole diameter. As the sensing distance increases, the coordinate prediction error of the target source gradually expands, but the prediction error of the target *x*-axis coordinates is generally lower than that of the *y*-axis coordinates, and with the increase in the sensing distance, the predicted values of the *y*-axis coordinates all show positive deviations. Therefore, it is shown that the least squares localization model based on the quasi-Newton method for predicting the perceived distance needs to increase the correction factor according to the perceived distance to be able to predict the perceived distance accurately.

**Figure 10:**
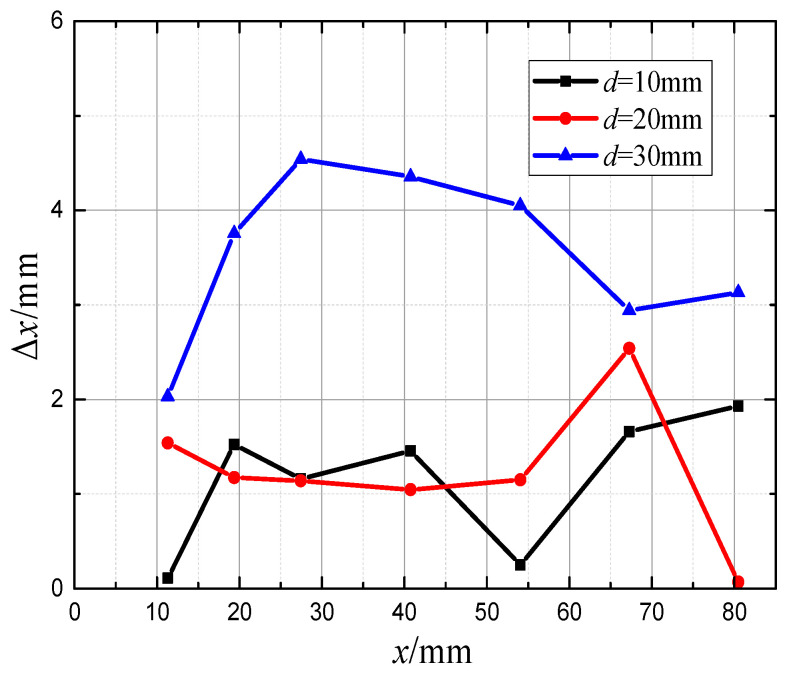
Prediction error of *x*-axis coordinates at different distances.

**Figure 11:**
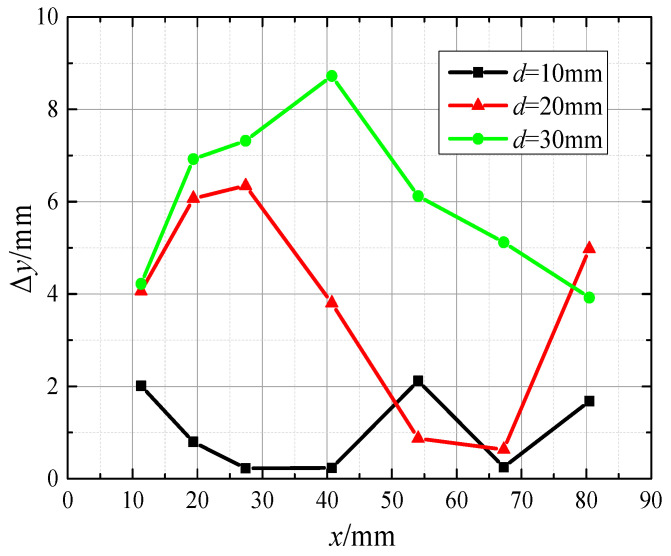
Prediction error of *y*-axis coordinates at different distances.

The analysis of the prediction error results for *x*-axis coordinates and *y*-axis coordinates is shown specifically in Figure 10 and Figure 11. As can be seen in Figure 10, the prediction errors for the *x*-axis coordinates are 1.24 mm and 3.54 mm when the perceived distances are d=20 mm and d=30 mm, with relative errors of 2.44% and 6.97%, respectively, and it can be seen that when the perceived distance is increased from d=10 mm to d=20 mm, the relative prediction error increases by only 0.14%. However, the relative prediction error increased by 4.53% when the perceived distance was increased from d=20 mm to d=30 mm. This suggests that there is an effective perceptual range for target localization based on bionic lateral line perception; the range is related to the intensity of the flow field disturbance of the target. Within this sensing range, the prediction error for the *x*-axis coordinates does not increase significantly as the sensing distance increases, and when the sensing range is exceeded, the prediction error for the *x*-axis coordinates increases rapidly.

**Figure 12:**
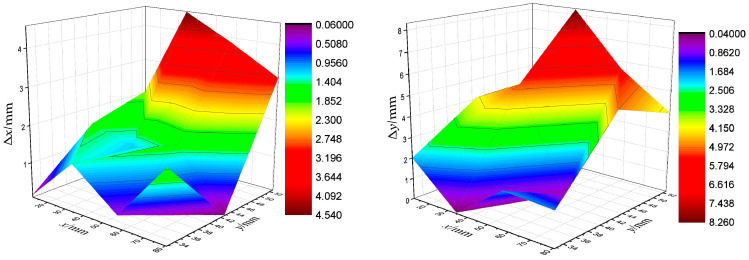
Distribution cloud of target localization error at different locations.

As can be seen in Figure 11, the prediction errors for the *y*-axis coordinates were 3.82 mm and 6.05 mm when the perceived distances were d=20 mm and d=30 mm, with relative errors of 7.52% and 11.91%, respectively. Meanwhile, the relative prediction error increased by 5.47% when the perceived distance increased from d=10 mm to d=20 mm. The relative prediction error increased by 4.39% when the sensing distance increased from d=20 mm to d=30 mm. It can be seen that the *y*-axis coordinate prediction error will increase with the increase in perceived distance; with the *x*-axis coordinate prediction error with the change rule of perceived distance, there are obvious differences, as shown in Figure 12.

It is clear from Figure 12 that at the same sensing distance, the localization errors obtained at different horizontal positions of the target source do not vary much, but as the perceived distance increases, the localization error in the vertical coordinate appears to increase significantly. This is because, due to the *x*-axis is the direction of water flow, the flow field distribution is relatively uniform; in a certain range of flow field signal, there is a certain robustness. The *y*-axis is the vertical direction of the water flow, the flow field disturbance signal is generally weaker, there is a greater degree of interference, and there will be linear weakening with the increase in the perceived distance, indicating that the positioning of the vertical coordinate is more likely to be interfered with; this also indicates the need to carry out fine compensation of the positioning error.

## 5. Conclusions and Outlook

In this paper, the SUBOFF model is used as the target source, and the bionic sideline sensing mechanism is adopted. An equivalent dipole model of the perturbation of the target flow field is constructed based on the potential flow theory, and the distribution function of the pressure coefficient with the coordinate position is established. A CFD numerical simulation method is used to collect the pressure field signal as the input information for flow field sensing. The location of the target source is predicted by least squares and quasi-Newton algorithms, and the prediction error is analysed in detail, which in turn enables the perception of target position, revealing the principle of target localization and tracking based on lateral line sensory signals in fish neural centres. The following conclusions were obtained:

(1) From the perspective of sensing the target, an equivalent dipole source model of the SUBOFF target is established based on the potential flow theory, and the pressure field generated by the equivalent dipole model is verified to match the flow signal generated by the original SUBOFF model using the CFD simulation method. It is shown that dipole sources are representative of general target sources and can be used to describe the flow field of an underwater moving target acting on a lateral line.

(2) A target source localization function is proposed based on the least squares and quasi-Newton methods, and the flow field environment sensing mechanism and localization function are simulated and verified. The results show that the near-field target position can be effectively predicted based on the flow field sensing signal, and the position prediction error is within 3% relative to the target diameter within a certain sensing range (d=20 mm in this paper).

(3) A bionic robotic fish model with a nonlinear lateral line sensing array is constructed and the applicability of the proposed target localization model is analysed. The results show that along the direction of the lateral line, the positioning error relative to the target diameter within the effective sensing range is less than 7%, while in the perpendicular direction, the relative positioning error increases with the increase in the dipole distance, and all of them show a positive deviation phenomenon. Therefore, this deviation can be modified by simply introducing an error correction mapping.

The current study assumes a steady-state flow field environment. In future research, it is necessary to strengthen the theoretical and methodological studies on the detection of dynamic targets in complex environments using bionic lateral line sensing technology, explore the intrinsic coupling mechanism between target motion states and flow field characteristics, and achieve the prediction of target motion intentions and the real-time positioning of position coordinates; the coordinate position correction function found in this paper is studied in depth to improve the target accuracy and robustness. Meanwhile, we will build the corresponding pool towing experimental environment in the follow-up work and verify the feasibility of underwater target recognition based on flow field signal features by constructing the principle prototype model.

## Figures and Tables

**Table 1:** Comparative data for submarine drag coefficient *C_d_*.

Comparative Parameters	Experimental Group	Simulation Group	Relative Error
$C_{d}\times 10^{3}$	2.716	2.681	1.29%

**Table 2:** Signal monitoring point coordinates.

Number	*S*_1_	*S*_2_	*S*_3_	*S*_4_
*X*-axis coordinate	11.31 mm	27.44 mm	54.05 mm	80.47 mm
*Y*-axis coordinate	15.33 mm	20.48 mm	22.68 mm	19.78 mm
normal angle	116.5°	100.5°	2.0°	10.5°

**Table 3:** Simulation model construction parameters.

Component	Parameters	Symbol	Numerical Value
Computational domain	Length × Width × Height	L×W×H	3500 × 1000 × 1000 mm
	Distance from water surface	*D*	500 mm
Vibrating ball	Small ball diameter	*a*	50.8 mm
	Vibration direction	*//*	Horizontal plane *X*-axis direction
	Vibration frequency	*f*	45 Hz
	Vibration amplitude	*s*	10 mm
Robotic fish model	Length	L	180 mm
	Number of sampling points	*N*_s_	4 × 2 rows
Signal acquisition	Time step	Δt	5 × 10^−4^ s
	Data length	*t*	20 s

## Data Availability

The original contributions presented in this study are included in the article. Further inquiries can be directed to the corresponding author.
